# The effects of social mindfulness and online interpersonal trust on college students’ online prosocial behavior

**DOI:** 10.3389/fpsyt.2025.1573345

**Published:** 2025-04-23

**Authors:** Jiajia Hao, Yinuo Liu, Xinran Ma, Shimiao Gong

**Affiliations:** ^1^ Faculty of Psychology, Tianjin Normal University, Tianjin, China; ^2^ Key Research Base of Humanities and Social Sciences of the Ministry of Education, Academy of Psychology and Behavior, Tianjin, China

**Keywords:** online prosocial behavior, social mindfulness, perceived prosocial impact, online interpersonal trust, contextual credibility

## Abstract

**Introduction:**

With the widespread adoption of the internet and social media, adolescents’ social interactions through online platforms have increasingly expanded. Adolescents’ prosocial activities in cyberspace not only serve as important indications of their socialization but also show how the internet and technology impact the new generation’s psychological adjustment and social integration patterns. Our study aimed to construct a moderated mediation model to explore the impact of social mindfulness on online prosocial behavior, as well as the mediating role of perceived prosocial impact and the moderating role of online interpersonal trust. Three experiments were designed based on the model to verify the applicability of the previously constructed model in real-world contexts.

**Methods:**

In Study 1, a cross-sectional study was conducted at a college in China, recruiting 328 college students using self-report questionnaires. The Social Mindfulness Self-Report Scale, the Online Prosocial Behavior Extension Scale (Chinese Version), the Perceived Social Impact Scale, and the Online Interpersonal Trust Scale were used. This study explored the relationship between social mindfulness and online prosocial behavior, the mediating effect of perceived prosocial impact, and the moderating role of online interpersonal trust. In Study 2, we adopted experimental research among 60 Chinese college students to explore the prosocial behavior of participants with different levels of social mindfulness in online contexts with varying levels of credibility.

**Results:**

(1) Social mindfulness positively predicted online prosocial behavior, with perceived prosocial impact serving as a partial mediator. Online interpersonal trust moderated the latter part of the mediation model. (2) Social mindfulness and contextual credibility positively predicted college students’ online prosocial behavior.

## 1 Introduction

In the digital era, people’s lifestyles, social patterns, and habits are undergoing unprecedented transformations. The behavioral patterns and values of young people—particularly college students, who represent the primary demographic of internet users—are profoundly influenced by the online environment. The internet not only expands opportunities for social interaction but also creates new platforms for the expression of prosocial behavior. Prosocial behavior, defined as actions that benefit others or society (1), manifests uniquely in online contexts as online prosocial behavior. Online prosocial behavior refers to voluntary acts in digital spaces that promote the well-being of online peers or foster positive relationships, without the expectation of external rewards (2, 3). Such behavior enhances individuals’ psychological well-being (4), contributes to societal welfare, and strengthens social cohesion. Distinct from offline prosocial behaviors, online prosocial behavior is characterized by virtual contexts, spatiotemporal flexibility, and the amplifying effects of social media. These unique attributes complicate the motives and influencing factors underlying online prosocial behavior. Investigating these mechanisms is critical for understanding adolescents’ digital-era behavioral patterns and psychological traits, as well as for guiding their constructive engagement in online prosocial acts to support healthy development.

Social mindfulness is defined as an individual’s ability to respect and perceive others’ states during interpersonal interactions, as well as to honor others’ choices by prioritizing their decision-making rights (5). Social mindfulness can be both a state and a trait (6). At the state level, it is activated by interpersonal relationships or situational factors; at the trait level, it represents a stable internal personality characteristic. This study specifically investigates social mindfulness at the trait level. Social cognitive theory (7) posits that individuals’ comprehension of others’ thoughts, emotions, and needs is closely tied to their social behaviors. The attentional sensitivity to others’ needs inherent in social mindfulness enhances prosocial behaviors (8). Van Doesum et al. (9) demonstrated that heightened social mindfulness correlates strongly with a prosocial value orientation. Similarly, Xie et al. (10) and Lv et al. (11) identified a positive association between social mindfulness and online altruistic behavior, highlighting its role as a key predictor of online prosocial behavior. Within China’s interdependent cultural framework, individuals are inclined to prioritize relational harmony with others (12), a tendency that may foster social mindfulness and amplify engagement in online prosocial behaviors. Thus, this study proposes Hypothesis 1: Social mindfulness positively predicts college students’ online prosocial behavior.

Perceived prosocial impact refers to the extent to which individuals perceive their actions to positively affect others. Existing research shows that perceived prosocial impact positively influences proactive behaviors (13). The perspective-taking capacity inherent in social mindfulness facilitates enhanced perception and comprehension of others’ affective states and viewpoints (14), which may enhance individuals’ perception of their behaviors’ positive impacts. According to the Feelings-as-information theory (FIT), subjective emotional states guide behavioral choices, with individuals basing subsequent actions on their feelings (15). When individuals recognize their behavior’s positive effects, this perceived prosocial impact may reinforce their motivation to engage in further prosocial acts. Collectively, the perceived prosocial impact may mediate the link between social mindfulness and online prosocial behavior. Thus, this study proposes Hypothesis 2: Perceived prosocial impact mediates the relationship between social mindfulness and online prosocial behavior.

Interpersonal communication in cyberspace occurs through virtual communities, enabling bidirectional and multidirectional interactions among strangers. The virtual, anonymous, and open nature of the internet, coupled with information uncertainty, complicates trust-building online, often leading individuals to doubt others’ intentions. Consequently, online interpersonal trust emerges as a critical factor influencing prosocial behavior (16, 17). Online interpersonal trust extends real-world relational trust into digital spaces, reflecting an individual’s expectation that others will honor commitments in online interactions (18). According to Social Exchange Theory, interpersonal trust involves positive expectations about others’ behaviors, which motivate individuals to engage in interactions through sharing, assisting, and collaborating (19). Studies demonstrate a significant positive correlation between online trust and altruistic behavior (20), which can be explained by reduced doubt, diminished psychological distance (21), and enhanced cooperation. Deng et al. (22) further link heightened trust to increased information-sharing. Self-determination theory (23, 24) posits that fulfilling individuals’ needs for autonomy, competence, and relatedness may motivate them to exert positive influences on others. Prior research (25) has found that in prosocial behavior, individuals tend to focus on demonstrating their capabilities. When individuals perceive that their prosocial actions generate positive impacts on others, they are more likely to recognize their abilities and values, thereby satisfying their competence needs and shaping a more positive self-perception. This self-perceived positive influence further reinforces subsequent prosocial behaviors. Meanwhile, online interpersonal trust may reflect the need for relatedness, as it involves the ability to build trust and connections with others. Enhancing individuals’ online interpersonal trust can strengthen their sense of belonging, encouraging more active engagement in online prosocial behaviors. The interaction between perceived prosocial impact and online interpersonal trust may create a synergistic cycle that amplifies online prosocial behaviors through a dual-path reinforcement mechanism grounded in the interplay of competence needs and relatedness needs. Thus, this study proposes Hypothesis 3: Online interpersonal trust moderates the relationship between perceived prosocial impact and online prosocial behavior.

Trust is a multidimensional concept encompassing both subjective and objective dimensions. At the subjective level, trust manifests as a stable personality tendency, namely online interpersonal trust; at the objective level, it is operationalized as context-dependent levels of trust (26). Synthesizing prior research, the credibility attributes of information (27), particularly authoritative information sources, have been shown to significantly shape individuals’ trust judgments. This context-dependent trust level activated by source characteristics may consequently modulate the manifestation of prosocial behaviors in digital environments. A critical inquiry thus arises: Do college students with different levels of social mindfulness exhibit differences in online prosocial behaviors when faced with online contexts of varying credibility? Thus, in the context of digital interconnectedness, it is essential to explore how social mindfulness and situational credibility influence college students’ online prosocial behaviors. From the perspective of social cognitive theory (7), social mindfulness—as an individual-level variable affecting online prosocial behaviors—interacts with situational credibility, an environmental factor. Social mindfulness is a spontaneously proactive prosocial trait (28). Individuals with high social mindfulness inherently possess stronger prosocial tendencies, and their intense intrinsic motivation makes them more likely to engage in prosocial behaviors across contexts with differing credibility. For those with low social mindfulness, their prosocial behaviors rely more on external environmental stimuli and guidance. Individuals are more willing to participate in prosocial behaviors in high-credibility contexts, whereas in low-credibility contexts, they may reduce such engagement due to distrust in others (29). Therefore, this study proposes Hypothesis 4: Social mindfulness and contextual credibility interact to influence college students’ online prosocial behaviors.

**Figure 1:**
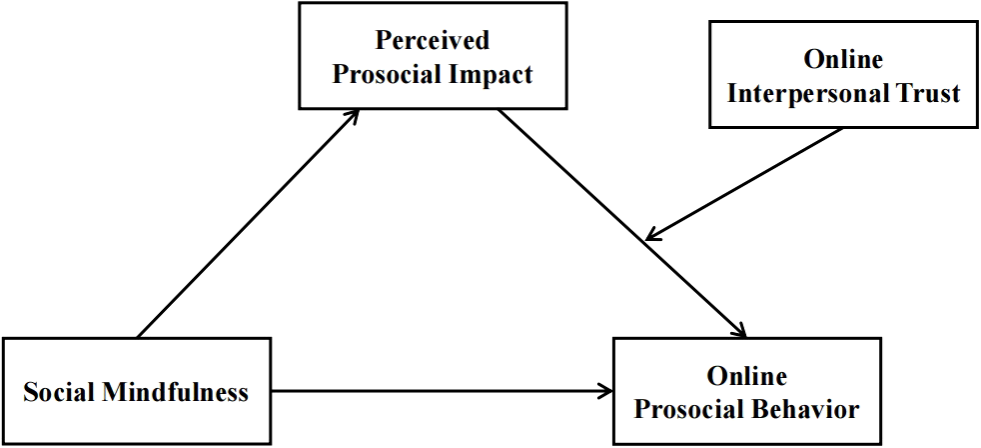
The hypothesized moderating mediation model.

This study investigates how personal traits (social mindfulness) and contextual factors (trust) influence online prosocial behaviors through two experiments. Based on Social Cognitive Theory, Feelings-as-Information Theory and Self-Determination Theory, study 1 employs a moderated mediation model (**Figure 1**) to examine interactions among social mindfulness, perceived prosocial impact, and online interpersonal trust. It focuses on individual differences in trust as a personality tendency. Study 2 manipulates contextual trustworthiness (high vs. low) to assess trust as an objective, situational variable. By controlling for social mindfulness and manipulating environmental trust levels, this experiment tests the combined effects of personal and contextual factors on online prosocial behaviors.

## 2 Study 1: The effect of social mindfulness on college students’ online prosocial behavior: a moderated mediation model

This study employed a questionnaire method to examine the relationship between college students’ social mindfulness and online prosocial behaviors, the mediating role of perceived prosocial impact, and the moderating role of online interpersonal trust.

### 2.1 Methods

#### 2.1.1 Participants

A total of 380 questionnaires were distributed. After excluding incomplete or insincere responses, 335 questionnaires were retained (retention rate: 88.2%). Further removal of extreme values yielded 328 valid responses (validity rate: 97.9%). Participants’ ages ranged from 17 to 27 years (*M* = 20.046, *SD* = 1.451). Detailed sample characteristics are presented in **Table 1**. The study was approved by the university’s ethics review board, and all participants provided voluntary participation after signing informed consent forms.

**Table 1:** Demographic variables (N=328).

Variables	Groups	*N*	Proportion
Gender	Male	101	30.8%
	Female	227	69.2%
Grade	Freshman	78	23.8%
	Sophomore	119	36.3%
	Junior	88	26.8%
	Senior	31	9.5%
	Postgraduate	12	3.7%

#### 2.1.2 Materials

##### 2.1.2.1 Online Prosocial Behavior Extended Scale (Chinese version)

Adapted from Van de Groep and Crone’s (30) Online Prosocial Behavior Scale-Extended (OPBS-E), this 13-item Chinese version assesses two dimensions: online emotional support and online activism. Responses were recorded on a 5-point Likert scale (1 = never, 5 = daily), with higher scores indicating greater frequency of online prosocial behaviors via digital platforms (e.g., smartphones, computers) in the past month. Cronbach’s α for this scale was 0.859.

##### 2.1.2.2 Social Mindfulness Self-Report Scale

The 17-item scale developed by Tian et al. (31) measures social mindfulness using a second-order four-factor structure: mindfulness/respect, modesty/respectability, tolerance/understanding, and positivity/openness. Responses were rated on a 5-point Likert scale (1 = strongly disagree, 5 = strongly agree). Higher scores reflect stronger social mindfulness. Cronbach’s α for this scale was 0.816.

##### 2.1.2.3 Perceived Prosocial Impact Scale

Lok and Dunn’s (32) 5-item self-report scale measures perceived behavioral impact on others using a 7-point Likert scale (1 = strongly disagree, 7 = strongly agree). Higher scores indicate a greater perceived positive impact of prosocial acts. Cronbach’s α for this scale was 0.881.

##### 2.1.2.4 Online Interpersonal Trust Scale

Ding and Shen’s (33) 9-item scale evaluates three dimensions: general trust, affective trust, and reliability trust in online contexts. Responses were recorded on a 5-point Likert scale (1 = completely disagree, 5 = completely agree), with items 6 and 9 reverse-scored. Cronbach’s α for this scale was 0.655.

#### 2.1.3 Statistical analysis

SPSS 26.0 was used to perform descriptive statistics and correlation analyses. The PROCESS (Model 4) tested the mediating role of perceived prosocial impact between social mindfulness and online prosocial behaviors. Model 14 further examined the moderating effect of online interpersonal trust on the latter half of the mediation pathway.

#### 2.1.4 Common method bias test

Self-report measures were used, which may introduce common method bias. Harman’s single-factor test revealed 10 factors with eigenvalues greater than 1, with the first factor explaining 21.3% of variance—below the 40% threshold (34), indicating no significant bias.

### 2.2 Results

#### 2.2.1 Descriptive statistics and correlation analysis

As shown in **Table 2**, social mindfulness, online prosocial behaviors, perceived prosocial impact, and online interpersonal trust were positively correlated (*r* = 0.232–0.583, *p* < 0.01).

**Table 2:** Descriptive statistics, correlation analysis for each variable (*N* = 328).

Variables	*M*±*SD*	1	2	3	4
1. Social mindfulness	68.253 ± 6.736	1.000			
2. Online prosocial behavior	46.442 ± 7.559	0.385**	1.000		
3. Perceived prosocial impact	27.238 ± 4.084	0.456**	0.583**	1.000	
4. Online interpersonal trust	27.610 ± 4.685	0.232**	0.284**	0.283**	1.000

**p<0.01, *p<0.05.

#### 2.2.2 Mediating effect of perceived prosocial impact

**Figure 2:**
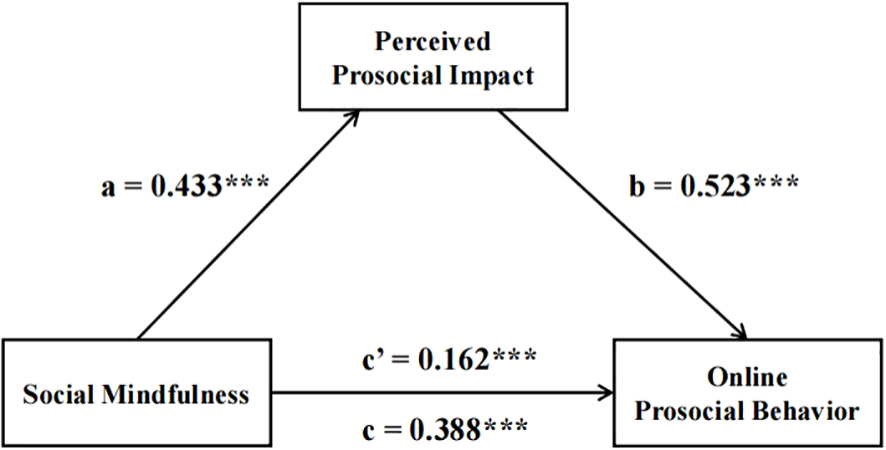
The mediating effect of social mindfulness and online prosocial behavior. ***p <0.001.

Standardized processing was applied to all variables. Using PROCESS Model 4 (controlling for gender and grade), social mindfulness significantly predicted online prosocial behaviors (*B* = 0.388, *SE* = 0.049, *p* < 0.001, *95% CI* [0.291, 0.486]). With perceived prosocial impact as a mediator, results are shown in **Figure 2**, social mindfulness predicted perceived impact (*B* = 0.433, *SE* = 0.047, *p* < 0.001, *95% CI* [0.340, 0.526]), which in turn predicted prosocial behaviors (*B* = 0.523, *SE* = 0.050, *p* < 0.001, *95% CI* [0.423, 0.622]). The direct effect of social mindfulness remained significant (*B* = 0.162, *SE* = 0.048, *p* = 0.001, *95% CI* [0.068, 0.257]). Mediation analyses indicated partial mediation (indirect effect = 0.226, *95% CI* [0.160, 0.298]), accounting for 58.2% of the total effect. Specific results are shown in **Table 3**.

**Table 3:** The mediating effect of perceived prosocial impact.

	Effect	*SE*	LLCI	ULCI	Proportion
Total effect	0.388	0.049	0.291	0.486	
Direct effect	0.162	0.048	0.068	0.257	41.8%
Indirect effect	0.226	0.035	0.160	0.298	58.2%

#### 2.2.3 Moderating effect of online interpersonal trust

**Figure 3:**
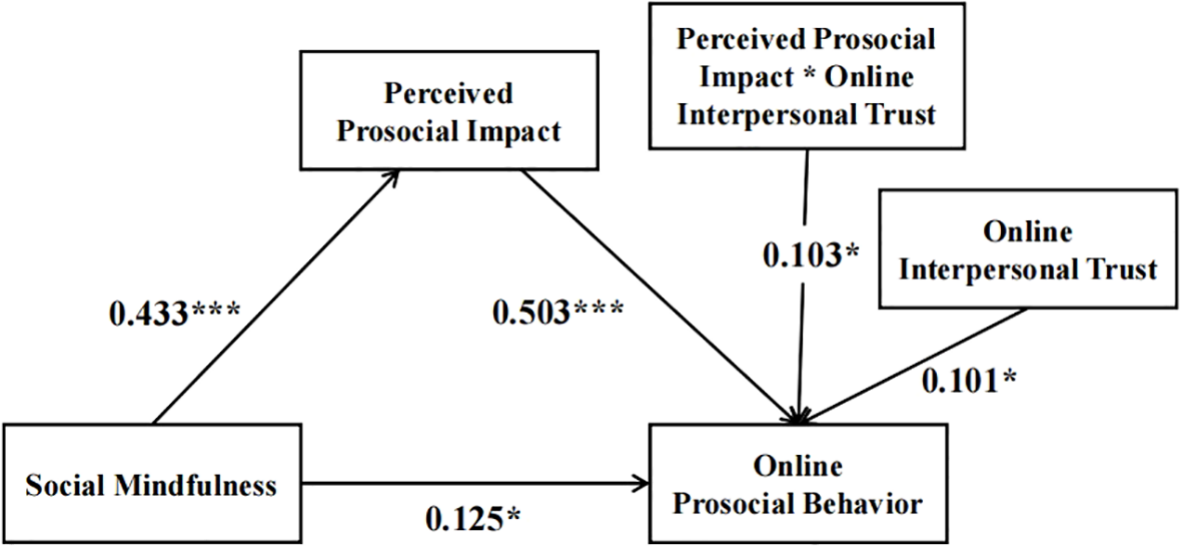
The moderated mediating effect of social mindfulness and online prosocial behavior. *p <0.05, ***p <0.001.

PROCESS Model 14 demonstrated that social mindfulness had a positive predictive effect on perceived prosocial impact (*B* = 0.433, *SE* = 0.047, *p* < 0.001). Furthermore, perceived prosocial impact exhibited a substantial predictive effect on online prosocial behavior (*B* = 0.503, *SE* = 0.051, *p* < 0.001). The interaction between perceived prosocial impact and online interpersonal trust significantly predicted prosocial behaviors (*B* = 0.103, *SE* = 0.048, *p* = 0.032), indicating that the latter stage of the mediation pathway—where trait social mindfulness exerts its effect on online prosocial behavior through perceived prosocial impact—was moderated by online interpersonal trust. Specific results are shown in **Table 4** and **Figure 3**.

**Table 4:** Moderated intermediation.

	Perceived prosocial impact			Online prosocial behavior		
	Effect	*SE*	*t*	Effect	*SE*	*t*
Social mindfulness	0.433	0.047	9.155^***^	0.125	0.049	2.571^*^
Perceived prosocial impact				0.503	0.051	9.869^***^
Online interpersonal trust				0.101	0.044	2.307^*^
Perceived prosocial impact×Online interpersonal trust				0.103	0.048	2.150^*^
Gender	0.027	0.097	0.274	0.269	0.087	3.102^**^
Grade	-0.006	0.042	-0.132	-0.052	0.039	-1.349
*R^2^*	0.208			0.400		
*F*	28.348^***^			35.656^***^		

***p<0.001, **p<0.01, *p<0.05.

**Figure 4:**
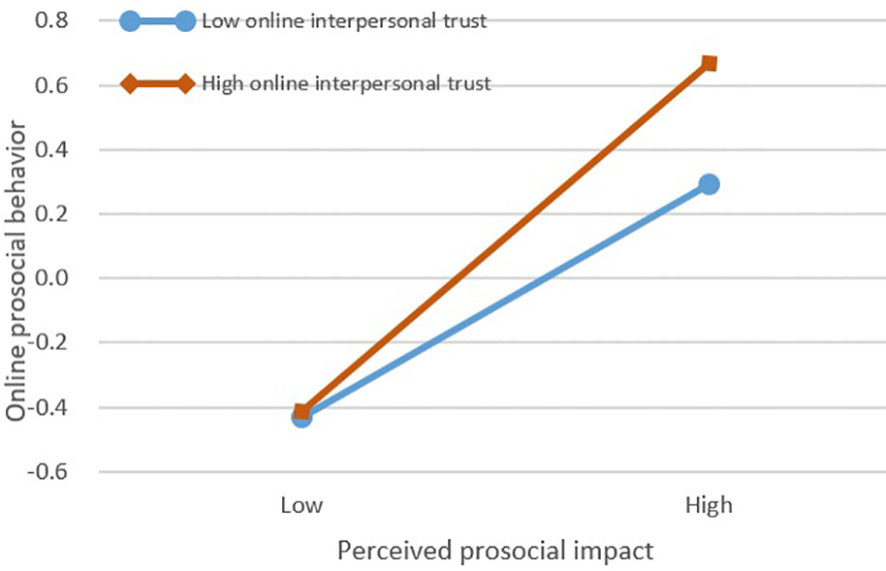
The moderating role of online interpersonal trust in the relationship between perceived prosocial impact and online prosocial behavior.

To explore how online interpersonal trust moderates the relationship between perceived prosocial impact and online prosocial behavior, participants were divided into high/low trust groups using validated measures, applying ±1 SD criteria from mean trust scores to create comparison groups. As shown in **Figure 4**, simple slope analyses revealed a differential predictive pattern: while perceived prosocial impact positively predicted college students’ online prosocial behavior in the low-trust group (*B* = 0.402, *SE* = 0.066, *p* < 0.001, *95% CI* [0.271, 0.533])., this predictive relationship was significantly amplified in the high-trust group *(B* = 0.603, *SE* = 0.072, *p* < 0.001, *95% CI* [0.462, 0.744]).

## 3 Study 2: The effects of social mindfulness and contextual credibility on college students’ online prosocial behavior

Building on Study 1’s moderated mediation model linking social mindfulness, perceived prosocial impact, and online interpersonal trust to online prosocial behaviors. Study 2 implemented a randomized controlled experimental design to validate the causal chain. However, because perceived prosocial impact involves individuals’ intrinsic evaluations of the social utility of their actions (e.g., “Does my behavior benefit others?”), its nature as a psychological construct posed challenges to direct experimental manipulation. Thus, Study 2 only explored the effects of social mindfulness and online contextual credibility on online prosocial behavior. The study integrated both the online emotional support and online activism subscales from the Chinese adaptation of the Online Prosocial Behavior Scale-Extended (OPBS-E) with conventional prosocial measures including online donations. Therefore, the dependent variables in this study: are (1) willingness to forward charity messages (measured in days), (2) number of recorded audio clips for charity projects, and (3) proportion of online donations. This integration aimed to comprehensively assess online prosocial behaviors.

### 3.1 Methods

#### 3.1.1 Participants

Following Wu’s (35) group screening criteria, participants were ranked based on their Social Mindfulness Self-Report Scale scores, ordered from highest to lowest. They were categorized into a high-social-mindfulness group (top 30%) and a low-social-mindfulness group (bottom 30%). Each group comprised 30 participants with a gender-balanced composition. The high-social-mindfulness group (age range: 18–21 years; *M* = 19.433, *SD* = 0.935) and the low-social-mindfulness group (age range: 18–23 years; *M* = 19.600, *SD* = 1.221) were then statistically compared. All procedures were approved by the university’s ethics review board.

#### 3.1.2 Experimental design

A 2 (social mindfulness: high vs. low) × 2 (contextual credibility: high vs. low) mixed design was employed. Social mindfulness served as a between-subjects factor, contextual credibility as a within-subjects factor, and dependent variables included the number of days to forward, recorded audio clips, and the proportion of their 10 RMB participation fee donated online.

#### 3.1.3 Materials

##### 3.1.3.1 Social Mindfulness Self-Report Scale

This was the same measure as Study 1.

##### 3.1.3.2 Forwarding punch card contexts

The experimental design incorporated two sets of posters for the Public Welfare Punch-Card Activity, featuring (A) *Help for Veterans in Difficulty* and (B) *Charity Warms Ten Thousand Homes*, with high- and low-credibility posters in each set. Drawing on Zheng et al. (36), credibility cues were manipulated across two conditions: high-credibility posters prominently displayed institutional authentication markers, including the Ministry of Civil Affairs (MCA) filing number and the program’s social credit code. Low-credibility posters retained only the charity appeal title, omitting supplementary trust-building elements. All other design elements (e.g., layout, color scheme) remained identical.

##### 3.1.3.3 Recorded contexts and online donation contexts

We developed four authentic websites simulating online prosocial behavior. These platforms included two modules: Read for You and Donate for Charity. All websites maintained identical content elements except for the placement of credibility cues.

The Read for You module featured two programs: (A) Read to Alzheimer’s Disease and (B) Read to Autism. Contextual credibility was manipulated through initiating organizations: the Alzheimer’s program was sponsored by either the high-credibility Alzheimer’s Disease Prevention Association or the low-credibility Dovatt’s Extraordinary Society; The autism program was sponsored by the official Beijing Disabled Persons’ Federation or the private Chandos Alliance. All audio recordings used 400-word excerpts from Wang Zengqi’s prose.

The Donation for Charity module included (A) The Power of an Egg and (B) Milk Powder for Babies in Distress. We control the credibility of the donation projects by setting up different initiating organizations: high-credibility institutions (Soong Ching Ling Foundation, China Children’s Charity Foundation) and low-credibility entities (CL Network, Infrared Express Coalition).

#### 3.1.4 Procedure

Subjects were invited through WeChat to participate in an experiment investigating “Willingness to forward public welfare punch-card activities”. Two sets of posters were distributed as image files, with the presentation order counterbalanced during distribution to mitigate order effects. Participants received the following instruction before the task: “You are invited to participate in a study investigating willingness to engage with a Public Welfare Punch-Card Activity. Specifically, we ask you to indicate how many days you would be willing to share two charitable posters on social media platforms to promote public awareness. You can choose not to participate, to participate for 1 day, 2 days, or 3 days.” Responses were recorded via WeChat using the following coding system: no response: 0, non-participation: 1, 1-day participation: 2, 2-day participation: 3, and 3-day participation: 4. After the task, participants were instructed to evaluate the perceived credibility of the content presented in the posters using a 7-point Likert scale (1 = “not at all believable”, 7 = “completely believable”).

The experiment was conducted in a behavioral laboratory. Four websites were displayed to participants according to group assignments using a Latin square design. Participants completed two sequential tasks. In the “Read for You” task, participants were instructed to browse initiative materials sequentially and select the “Select Fragment Button” to specify the number of prose segments (0–5) they would read aloud for individuals with Alzheimer’s disease and autism. Each 400-word segment required approximately 90 seconds to complete, with participants free to self-determine the recording sequence. After finalizing their selections, participants accessed assigned materials via the “Read Aloud Clip Material” interface to initiate audio recordings. Upon completion, progression to the “Donate to the Community” task was achieved by clicking the “Next Page” button. In this section, participants reviewed the program description of public welfare donation initiatives and allocated a self-determined percentage of their participation compensation (0%, 20%, 40%, 60%, 80% or 100%) to support individuals in need through charitable programs. Subsequently, participants were required to independently assess the credibility of each task on the website. For this assessment, a 5-point Likert scale (1 = “very unbelievable”, 5 = “very believable”) was employed. Finally, participants were required to click on the questionnaire to complete it. Final instructions specified that donation percentages would not be deducted from actual payments, and all participants received a 10 RMB compensation.

#### 3.1.5 Statistical analysis

Data were analyzed using Generalized Estimating Equations (GEE) in SPSS 26.0. Participant ID was specified as the subject variable, with social mindfulness group (high vs. low) and contextual credibility (high vs. low) as independent variables. A linear model assessed effects on retweet days, recorded audio clips, and donation proportion, controlling for grade level.

### 3.2 Results

#### 3.2.1 Willingness to forward

##### 3.2.1.1 Operational check of contextual credibility

The credibility manipulation was validated through comparative analysis. Results revealed significant differences between the two posters in Group A materials for contextual credibility (*t*(29) = 6.858, *p* < 0.001). The Group B materials similarly demonstrated significant divergences in contextual credibility (*t*(29) = 7.629, *p* < 0.001).

##### 3.2.1.2 Analysis of forwarding days

As presented in **Table 5**, GEE analysis revealed a significant main effect of the social mindfulness group (Wald *χ*²(1) = 4.358, *p* = 0.037), demonstrating increased forwarding frequency in the high-social-mindfulness group. Furthermore, a statistically significant main effect of contextual credibility was observed (Wald *χ*²(1) = 23.438, *p* < 0.001), indicating more forwarding days in high-credibility contexts. However, the interaction between social mindfulness and credibility did not reach statistical significance (Wald *χ*²(1) = 0.938, *p* = 0.333). No significant main effect of grade level was detected (Wald *χ*²(1) = 0.022, *p* = 0.883).

**Table 5:** GEE analysis of willingness to forward charity punch cards (*N*=60).

Variables	Wald*χ²*	*df*	*p*
Social mindfulness	4.358	1	0.037
Contextual credibility	23.438	1	<0.001
Social mindfulness × Contextual credibility	0.938	1	0.333
Grade	0.022	1	0.883

#### 3.2.2 Recording behavior

##### 3.2.2.1 Operational check of contextual credibility

The contextual credibility manipulation demonstrated robust validity across experimental conditions. For Group A materials, high-authority initiating organizations elicited significantly greater credibility than low-authority counterparts (*t*(30) = 9.992, *p* < 0.001), with parallel effects observed in Group B (*t*(28) = 9.628, *p* < 0.001).

##### 3.2.2.2 Analysis of recorded audio clips

As shown in **Table 6**, the significant main effects of the social mindfulness group (Wald *χ*²(1) = 25.492, *p* < 0.001), with the high-social-mindfulness group demonstrating substantially more recorded audio clips. A significant main effect of contextual credibility was also observed (Wald *χ*²(1) = 56.842, *p* < 0.001), indicating elevated recording propensities in high-credibility contexts. No significant main effect emerged for grade level (Wald *χ*²(1) = 2.216, *p* = 0.137).

**Table 6:** GEE analysis of recording behavior (*N*=60).

Variables	Wald*χ²*	*df*	*p*
Social mindfulness	25.492	1	<0.001
Contextual credibility	56.842	1	<0.001
Social mindfulness × Contextual credibility	25.263	1	<0.001
Grade	2.216	1	0.137

A significant interaction (Wald *χ*²(1) = 25.263, *p* < 0.001) showed: that within the low social mindfulness group, college students selected significantly more audio clips in high-credibility contexts compared to low-credibility contexts (*p* < 0.001), with mean difference of 1.000 segments. In the high social mindfulness group, while a statistically significant difference in clip selection across credibility contexts was observed (*p* = 0.021), the high-credibility context exhibited merely a 0.200-segment elevation.

#### 3.2.3 Online donation behavior

##### 3.2.3.1 Operational check of contextual credibility

The manipulation check demonstrated significant credibility differences for both donation materials: Material A (*t*(31) = 8.783, *p* < 0.001) and Material B (*t*(27) = 10.510, *p* < 0.001), with campaigns initiated by high-authority organizations perceived as more credible than low-authority organizations.

##### 3.2.3.2 Analysis of the amount of donations

The results shown in **Table 7** revealed a significant main effect of the social mindfulness group (Wald *χ*²(1) = 3.996, *p* = 0.046), with higher donation amounts in the high social mindfulness group. A significant main effect of contextual credibility emerged (Wald *χ*²(1) = 12.658, *p* < 0.001), showing greater donations in high-credibility contexts versus low-credibility contexts. No significant main effect of grade level was found (Wald *χ*²(1) = 1.253, *p* = 0.263).

**Table 7:** GEE analysis of online donation behavior (*N*=60).

Variables	Wald *χ²*	*df*	*p*
Social mindfulness	3.996	1	0.046
Contextual credibility	12.658	1	<0.001
Social mindfulness × Contextual credibility	3.359	1	0.067
Grade	1.253	1	0.263

The interaction between social mindfulness and credibility approached marginal significance (Wald *χ*²(1) = 3.359, *p* = 0.067). Pairwise comparisons showed no significant difference in donation amounts was observed between high- and low-credibility contexts among college students in the high social mindfulness group (*p* = 0.143). Conversely, students in the low social mindfulness group donated significantly more in high-credibility contexts compared to low-credibility contexts (*p* = 0.001).

## 4 Discussion

### 4.1 Moderated mediation between social mindfulness and online prosocial behavior

Study 1 demonstrates a moderated mediation model in which social mindfulness influences online prosocial behavior through perceived prosocial impact, with online interpersonal trust acting as a contextual moderator. The results substantiate that social mindfulness directly and positively predicts online prosocial behavior, thereby confirming Hypothesis 1. These results align with extant empirical evidence showing positive correlations between social mindfulness and prosocial behavior (10, 37). Moreover, Manesi et al. (38) identified social mindfulness as a critical predictor of donation behaviors following Typhoon Haiyan in the Philippines. Individuals with heightened social mindfulness typically endorse prosocial value systems (9, 39), which amplify their altruistic motivations and energize sustained engagement in benevolent actions.

Mediation analysis revealed that perceived prosocial impact significantly mediates the relationship between social mindfulness and collegiate online prosocial behavior, thus validating Hypothesis 2. Previous studies have indicated a significant positive correlation between social mindfulness and perspective-taking ability (9);. Individuals with high social mindfulness tend to actively pay attention to others’ needs and engage in in-depth thinking from others’ perspectives. This cognitive orientation makes them more likely to acutely perceive the positive impacts of their own behaviors, thereby enhancing their perception of the value of these behaviors. According to social cognitive theory (7), there is a close connection between an individual’s social cognitive structure and behavioral patterns, with cognitive structure playing a key role in the manifestation of social behaviors. An individual’s perception of others’ positive social behaviors may act as an intrinsic driving factor, stimulating their willingness to engage in more prosocial behaviors, thereby creating a positive social incentive effect. Therefore, in online environments, individuals with high levels of social mindfulness are more likely to keenly perceive others’ needs, deeply understand the positive effects of their helping behaviors on others, and consequently exhibit more frequent and active prosocial behaviors.

The study further identified online interpersonal trust as a significant moderator of the relationship between perceived prosocial impact and collegiate online prosocial behavior, confirming Hypothesis 3. Specifically, online interpersonal trust amplifies the impact of perceived prosocial impact on collegiate online prosocial behavior. Among collegiate students with elevated online interpersonal trust, perceived prosocial impact exerts a stronger positive predictive effect on prosocial behavior This moderating pattern aligns with cost-benefit theory (40), which conceptualizes behavioral decisions as outcomes of cost-benefit evaluations. Within this theoretical framework, perceived prosocial impact represents cognitive appraisals of behavioral benefits (e.g., “My contributions meaningfully improve others’ welfare”), while online interpersonal trust becomes a key socio-cognitive variable that moderates this cognitive trade-off by reshaping individuals’ evaluation of behavioral costs. Specifically, when individuals exhibit higher levels of trust in online interpersonal interactions, their sensitivity to potential behavioral costs (e.g., risk exposure, resource depletion, or exploitation likelihood) diminishes, whereas their anticipation of prosocial behavioral benefits (e.g., confidence in meaningfully enhancing others’ well-being) intensifies. This “low-cost, high-benefit” cognitive schema facilitates a more efficient conversion of perceived prosocial impact into tangible behavioral enactment, resulting in a stronger positive predictive relationship between perceived prosocial impact and online prosocial behavior among high-trust individuals.

In summary, the interplay between social mindfulness, perceived prosocial impact, and online interpersonal trust elucidate the psychological mechanisms underlying college students’ online prosocial behaviors. These findings, partially empirically validated in Study 2, demonstrate that both social mindfulness and online contextual credibility (the objective dimension of trust) significantly enhance college students’ online prosocial behaviors. Specifically, participants with high social mindfulness exhibited significantly more prosocial behaviors in digital environments compared to the low-social-mindfulness participants. Furthermore, online prosocial behaviors were markedly greater in high-credibility contexts than in low-credibility contexts. The findings of this study advance a theoretical understanding of digital-era social dynamics and offer practical insights for fostering prosocial engagement in virtual environments.

### 4.2 Experimental study of social mindfulness and contextual trustworthiness on college students’ online prosocial behavior

Study 2 experimentally validated the effects of social mindfulness and contextual trustworthiness on online prosocial behavior through behavioral tasks involving charity information forwarding, project recording, and donations.

The results revealed significant main effects of social mindfulness and contextual trustworthiness across all three behavioral indicators. Participants with high-social mindfulness exhibited substantially greater willingness to forward charity information, higher recording frequency, and larger donation amounts compared to the low-social mindfulness group. Contextual trustworthiness similarly enhanced prosocial engagement: high-trust contexts yielded greater forwarding willingness, recording frequency, and donations than low-trust contexts. These findings extend prior work by demonstrating that contextual trust facilitates prosocial behavior not only in offline interpersonal interactions but also in online environments characterized by anonymity and unfamiliarity.

Notably, grade level showed no significant association with online prosocial behavior. This null effect may stem from the relative homogeneity of the college student sample in cognitive development. The anti-fraud and other propaganda carried out by the school covers all grades, which makes the whole group’s trust in online information have a certain degree of homogeneity.

For behavioral indicators, the nonsignificant interaction between social mindfulness and trustworthiness in forwarding willingness suggests trait stability across trust contexts. Furthermore, The relatively small sample size may also compromise the reliability of the findings regarding forwarding willingness. However, recording frequency displayed a significant interaction: low-social mindfulness participants were more sensitive to trust variations compared to high- individuals. Individuals in the low-social mindfulness group tended to interpret contextual situations through a more negative lens. Particularly in low-trust contexts, they exhibited heightened skepticism toward situational authenticity and remained vigilant about information credibility, resulting in cautious decision-making regarding prosocial behavioral engagement. This wariness amplified their sensitivity to behavioral choices across trust conditions. Individuals in the high-social mindfulness group exhibit a more positive and benevolent demeanor when confronted with identical situations. Even in low-credibility contexts, they are more inclined to engage in prosocial behaviors. This tendency may not solely rest on the credibility of contextual information but rather stems more from their inner goodness and trust in others. Even when they deem a situation as untrustworthy, they remain willing to uphold kindness and choose to assist others.

In donation behaviors, high-social mindfulness participants showed a marginally significant difference in contribution amounts across trust contexts, aligning with the conceptualization of social mindfulness as “small-cost gestures” (5). The donation program entails a fee of 5 RMB per item, offering a range of contribution percentages including 0%, 20%, 40%, 60%, 80%, and 100%. With the relatively small monetary amounts involved, this donation behavior aligns more closely with the concept of social mindfulness, representing actions of little to no effort. Consequently, it may evoke less sensitivity to contextual credibility.

### 4.3 Implications and limitations

This research comprises two interrelated studies. Study 1 establishes a moderated mediation model of perceived prosocial impact, incorporating online interpersonal trust as a moderator based on theoretical foundations and prior empirical evidence, to investigate the mechanisms through which social mindfulness facilitates online prosocial behaviors. Building upon these findings, Study 2 experimentally validates partial pathways within the proposed conceptual framework. The findings advance understanding of the relationship between social benevolence and college students’ online prosocial engagement. This study enriches the research field of online prosocial behavior and guides students’ proper online actions and psychological health development.

The findings of this study provide important implications for developing intervention strategies to enhance online prosocial behavior among college students. First, it is crucial to emphasize students’ online prosocial behaviors and focus on feedback regarding the perceived impact of these actions. This study reveals that online prosocial behaviors are prevalent among university students, primarily manifesting through emotionally supportive expressions such as liking, commenting, and sharing content. Therefore, educators should cultivate students’ awareness of both experiencing others’ online prosocial behaviors and consciously expressing their own prosociality in digital contexts. As the principle “actions speak louder than words” demonstrates, practical efforts and tangible behaviors often convey greater social warmth and efficacy than mere intentions. Second, fostering “small acts of kindness” and cultivating social mindfulness should be prioritized. The study demonstrates that high levels of trait social mindfulness significantly enhance online prosocial behaviors. Consequently, educators can create supportive learning environments that emphasize the expression of social skills, encourage peer interactions and collaborations, and promote the concept of “effortless helping.” Expressing kindness toward peers serves as an effective intervention for moral development. Integrating benevolence education into ethics curricula through dedicated modules, guiding students to experience and share social mindfulness, and enhancing their awareness of receiving and expressing goodwill can facilitate the transformation of knowledge into action. This approach effectively promotes the transmission of prosocial behaviors among individuals and their adaptation to online contexts.

This study has several methodological constraints. First, data collection approaches and measurement dimensions show limitations. Social mindfulness and online interpersonal trust were measured only by self-report questionnaires, prone to social desirability bias. Also, social goodwill measurement focused on the trait level. Future research could combine localized social mindfulness paradigms for state-level measurement including implicit trust measures to enhance reliability and explore how personal and environmental factors jointly affect online prosocial behavior, building a stronger theoretical model. Second, Study 2 didn’t include the mediator of perceived prosocial impact. Future work could use scenario simulation or priming to activate this mediator or adopt a longitudinal design for a more robust temporal mediation model. Third, the selected online prosocial behavior indicators lack adequate interactivity metrics. Future studies might integrate advanced page programming or dummy subject interaction to better simulate real-world online prosocial scenarios, enhancing behavioral interactivity and real-time data collection.

## Data Availability

The raw data supporting the conclusions of this article will be made available by the authors, without undue reservation.
